# Histologic and histomorphometric assessment of eggshell-derived bone graft substitutes on bone healing in rats

**DOI:** 10.4317/jced.50968

**Published:** 2013-02-01

**Authors:** Ahu Uraz, Sibel E. Gultekin, Burcu Senguven, Burcu Karaduman, Ilke P. Sofuoglu, Selcen Pehlivan, Yilmaz Capan, Kaya Eren

**Affiliations:** 1 Department of Periodontology, Gazi University Faculty of Dentistry, Ankara, Turkey

## Abstract

Objective: The objective of this study was to histologically and histomorphometrically evaluate the efficacy of the new formulations of eggshell-derived calcium carbonate in rats. Study Design: The study was conducted on 30 adult male rats. Four standardized and circular intrabony defects were created in the both maxilla and mandibula of each animal. Three different graft materials were prepared as follows: 1) Material A: Eggshell-derived calcium carbonate combined with carrageenan gel, 2) Material B: Eggshell-derived calcium carbonate combined with xanthan gum gel, and 3) Material C: Eggshell-derived calcium carbonate powder. The right mandibular defect sites were grafted with Material A in all animals, and defects on the left were grafted with Material B. Defects on the right side of maxilla were received Material C in all animals, and all left maxillary defects were remained untreated as controls. The animals were sacrificed either postoperatively on the 15th day, postoperatively on the 30th day or postoperatively on the 45th day. Histomorphometric measurements were made of the areas of newly formed bone, necrotic bone, fibrous tissue and residual graft material. Results: Material A exhibited the highest level of osteoid formation followed by Material B and Material C on the 45th day. In terms of osteoid formation, statistically significant differences were observed between graft materials and controls at 45th day compared to 15th and 30th day (p<0.05). Conclusions: Eggshell-derived graft substitutes in both gel and powder forms are biocompatible materials which may have the potential to enhance the new bone formation.

** Key words:**Bone graft material, bone defects, eggshell, histopathological evaluation, rat.

## Introduccion

The regeneration of supportive periodontal tissues after destructive periodontal diseases has become one of the primary objectives of periodontal therapy. To accomplish this goal various bone replacement graft materials have been extensively used with varying degrees of success (1-7)

Autogenous bone grafts, harvested from intraoral sites, have been accepted as the gold standard graft material due to their excellent biocompatible and osteogenic properties. The requirement for a wider or second surgery, unreliable graft incorporation, tooth ankylosis, and root resorption are the disadvantages of using autogenous bone grafts (1,2,7,8) Alternative biomaterials have been investigated to eliminate these problems. In recent years, natural bone substitutes, particularly coral skeleton composed of calcium carbonate (CaCO3), have been used as a bone graft substitute in the management of periodontal lesions. Coralline CaCO3, which is both fully resorbable and biocompatible, has been reported to have advantages in bone healing (9-12).

Avian eggshell with a mineral composition similar to that of coral (95 %) has been studied as a bone graft substitute in the oral cavity (13-15). Eggshell structure is well documented in the literature. Calcium, phosphorus, magnesium and sodium are major inorganic constituents of the avian eggshell. Eggshell powder is a safe and biodegradable material that is easy to obtain as well. Clinical and experimental studies have shown a number of positive effects of eggshell powder on bone metabolism. These features present qualify eggshell powders as a worthwhile bone substitute candidate (14-18) Furthermore, eggshell-derived CaCO3 has been demonstrated to be efficacious as a biocompatible and osteoconductive biomaterial in several experimental animal studies (19-22).

Although eggshell has been proposed and tested as a graft material previously, currently, very limited information available on the use of this material in different forms; to our knowledge no published histopathological studies are available. The objective of this study was to histologically and histomorphometrically evaluate the efficacy of the new formulations of eggshell-derived CaCO3 in rats.

## Material and Methods

- Subjects 

All animal procedures were approved by the Scientific and Ethics Committee of the Medical Faculty of Gazi University. A total of thirty male Sprague-Dawley rats (12 weeks old), each weighing 250-300 g, were obtained from the Experimental Animals Research and Application Center of the Medical Faculty of Gazi University. All of the necessary medical screenings on the test subjects, both before and following the operation, were carried out in the same units.

- Preparation of Hen’s Eggshell as a Graft Material

Hen eggshells were purchased from a local market. Eggshell powders were prepared as described previously.14 The eggs were rinsed with sterile distilled water. After the egg’s contents were poured, the outer and inner shell membranes of the eggs were carefully removed with forceps. The shells were crushed and sieved until particles (particle diameter, 1mm; porosity, 75%) were obtained. The powdered eggshells were rinsed three times in sterile distilled water with continuous agitation for 1 hour and at room temperature. They were then was autoclaved at 136° C for 18 minutes.

Powdered eggshells were combined with a gel matrix containing carrageenan or xanthan gum, both of which are well-known and documented materials used frequently in the pharmaceuticals industry to stabilize compounds (23,24).

Three different graft materials were prepared as follows.

1. Material A: Eggshell-derived calcium carbonate combined with carrageenan gel

2. Material B: Eggshell-derived calcium carbonate combined with xanthan gum gel

3. Material C: Eggshell-derived calcium carbonate powder.

Material A was prepared with 400 mg of eggshell-derived calcium carbonate powder and 200 mg of carrageenan (Sigma-Aldrich USA). Eggshell-derived calcium carbonate powder (400 mg) was incorporated into xanthan gum (200 mg) (Sigma, USA) in order to prepare Material B. Both mixtures were stirred with a metal rod in a beaker at 50 ºC until the gel was completely formed. The prepared gels were stored at -30 ºC, whereas eggshell-derived calcium carbonate powder (particle diameter, 1 mm; porosity, 75%) was stored at room temperature in 5 cc sterile vials until implantation.

- Surgical Procedure

Surgery was performed under general anesthesia with gluteal intramuscular injections of a combination of Ketamine HCl (Ketanes®, Alke, İstanbul, Turkey) and Xylazine (Rompun®, Bayer, Leverkusen, Germany) at a dose of 50 mg/kg and 5 mg/kg body weight, respectively. The operative site was shaved and cleaned with 10% povidone-iodine solution. After shaving, the surgical sites were exposed with an incision through the skin and periosteum. The dissection was carried out through the subcutaneous and muscle layers, followed by the careful dissection of the periosteum. Under constant copious irrigation with saline, a slowly rotating stainless steel trephine burr (ISO 0483, MK-BH26; MIS Implant Technologies®, Shlomi, Israel) was used to create 4 mm in diameter and depth standardized and circular intrabony defects in both the maxilla and mandible. In order to evaluate the efficacy of the eggshell-derived graft material in different gel matrices, four intrabony defects were created in each animal. The right mandibular defect sites were grafted with Material A in all animals, and defects on the left were grafted with Material B. Defects on the right side of maxilla were received Material C in all animals, and all left maxillary defects were remained untreated as controls.

A total of 0.5 cc of bone graft material was injected into each defect. After graft placement, the soft tissues were replaced and inner tissue flaps were sutured by layers with 3-0 silk sutures (Dogsan®, Trabzon, Turkey).

- Specimen Preparation and Histomorphometric Analysis

The animals were sacrificed either postoperatively on the 15th day (group 1), postoperatively on the 30th day (group 2) or postoperatively on the 45th day (group 3) using an intravenous overdose of a combination of keta-mine HCl and Xylazine. Thirty animals were randomly divided into three groups of 10-animals each.

The jaw bones were removed and en bloc biopsies were taken from the operated areas. All histological procedures and evaluations were carried out at the Department of Oral Pathology, Faculty of Dentistry, Gazi University. The specimens, along with the surrounding soft tissues, were fixed with 10% buffered formalin solution. All materials were decalcified with 10% formic acid for three weeks, which was followed by washing under tap water overnight. After dehydration and paraffin embedding, the specimens were serially cut into 5-µm-thick sections and stained with routine haematoxylin and eosin (H&E).

Histomorphometric analysis was performed to quantify the relative amounts of the tissue types in each defect under light microscopy (Leica® DM 4000, Germany) by using an image analysis system (Leica® QWIN3.2 Software). The following histomorphometric measurements were made: Dimensions of total defect area, newly formed bone (NFB), necrotic bone (NB), fibrous tissue (FT) and residual graft material (RG). Percentages of values also were obtained in relation to the total defect area.

Newly Formed Bone (NFB%) = New bone area / Total defect area x 100

Necrotic bone (NB%) = Necrotic bone area / Total defect area x 100

Fibrous tissue (FT%) = Fibrous tissue area / Total defect area x 100

Residual graft material (RG%) = Residual graft material area / Total defect area x 100

- Statistical analysis

All data were analyzed using the statistical package SPSS version 16.0 (SPSS Inc., Chicago Illinois, USA). Two-way repeated-measures ANOVA were used to determine differences between the materials over the study time period. Values of p<0,05 were considered to be statistically significant.

## Results

Postoperative healing was uneventful during the experimental period. There were no foreign body reactions to the materials.

- Histological Findings

**Figure 1:**
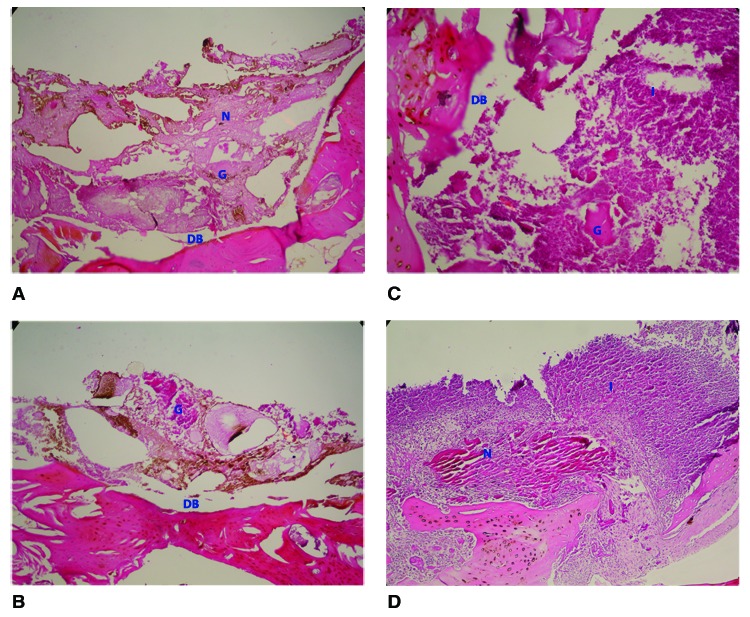
Histological findings at 2 weeks. (A) Material A group (H&E, x100); (B) Material B group (H&E, x100), (C) Material C group (H&E, x100); (C) Control defect (H&E, x40). N: Necrotic tissue; G: Remnants of graft material; DB: Defect base; I: Inflammation.

15th day findings:

All defects were mainly filled with fibrous connective tissue predominantly seen in control defects and defects filled with material C. In all samples, prominent and dense inflammation on fibrous connective tissue was seen. Remnants of graft material were embedded within the newly formed connective tissue in all experimental defects. No obvious new bone formation was seen. Material A showed very limited bone formation, however, there was no statistically significant difference compared to the other materials (p>0,05). Necrotic bone was evident in all experimental defects and was particularly marked in the control defects (Fig. 1).

**Figure 2:**
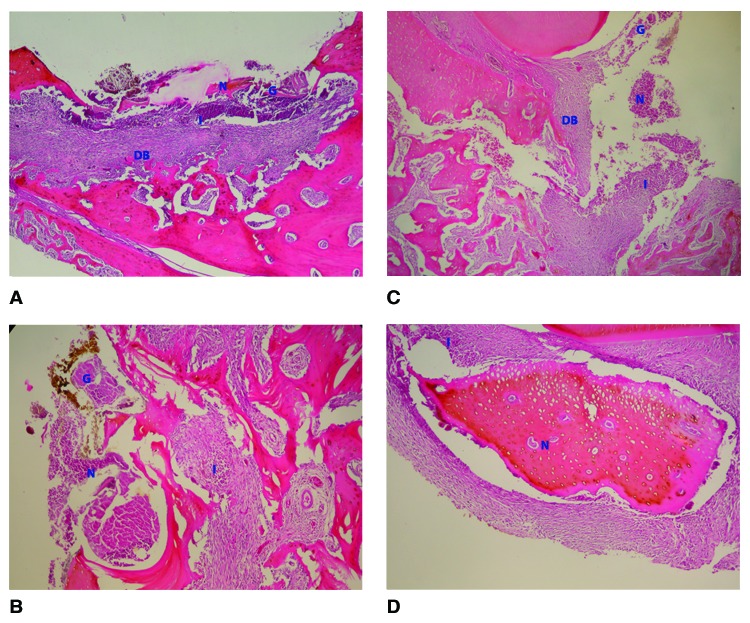
Histological findings at 4 weeks. (A) Material A group (H&E, x40); (B) Material B group (H&E, x100), (C) Material C group (H&E, x40); (C) Control defect (H&E, x40). N: Necrotic tissue; G: Remnants of graft material; DB: Defect base; I: Inflammation.

30th day findings:

Increased bone formation and decreased amounts of necrotic bone were observed compared with at 4 weeks of healing. New bone formation with very little remnants of graft materials was seen in all samples. Inflammation became moderate at 30 days (Fig. 2).

**Figure 3:**
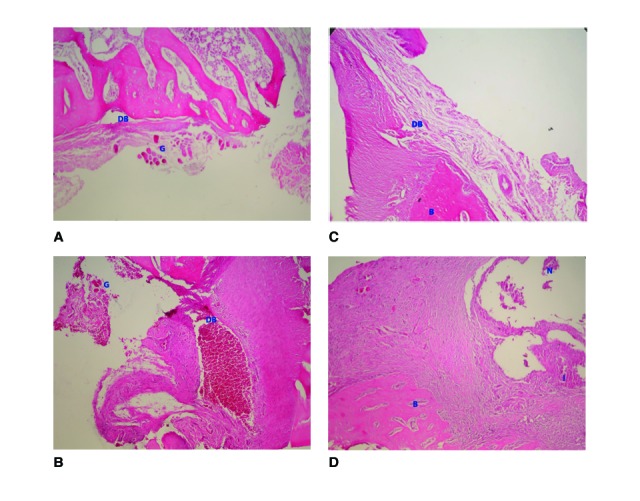
Histological findings at 6 weeks. (A) Material A group (H&E, x40); (B) Material B group (H&E, x40), (C) Material C group (H&E, x100); (C) Control defect (H&E, x40). G: Remnants of graft material; DB: Defect base; B: Bone; N: Necrotic tissue; I: Inflammation.

45th day findings:

An intensive new formation of bone was noticed in all experimental samples and inflammation was completely absent at 45 days. Minimal amounts of necrotic bone were seen in all samples. Complete defect healing occured in the specimens treated with the combination of calcium carbonate and carrageenan. No remnants of the graft materials were noted in any of samples. More connective tissue was found in control group after 45 days (Fig. 3).

- Histomorphometric Analysis/ Qualitative Histological Findings

**Figure 4:**
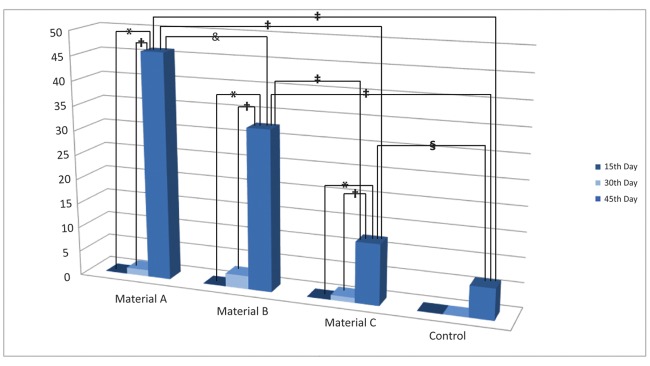
The percentage of newly formed bone (mean) in relation to the evaluation times in biopsy specimens is shown. *Statistically significant (p=0.02), † Statistically significant (p=0.010), ‡ Statistically significant (p=0.000), § Statistically significant (p=0.038), & Statistically significant (p=0.014.)

In terms of osteoid formation, statistically significant differences were observed between graft materials and control at 45th day (p=0.000) (p=0.038) (Material A; 46.15±0.24, Material B; 32.64±0.41, Material C; 12.31±0.39, Control; 6.29±1.01). All materials showed significantly higher bone formation on the 45th day than 15th and 30th day (p=0.02) (p=0.010). Material A exhibited the highest level of osteoid formation followed by Material B and Material C on the 45th day (46,15%, 32,64% and 12,31%, respectively) (p=0.000) (p=0.014). There was an increase in osteoid formation in all defects between 15 (Material A; 0.00±0.00, Material B; 0.00±0.00, Material C; 0.00±0.00, Control; 0.00±0.00) and 30 days (Material A; 1.29±0.48, Material B; 2.68±0.38, Material C; 0.99±0.97, Control; 0.00±0.00) without statistical significance (p>0.05) (Fig. 4).

On the 15th day, Material A (2.77±0.56) and B (3.06±0.90) produced statistically significantly lower amounts of necrotic bone than from Material C and control (p=0.00) (p<0.001). There were significant differences in the percentage of necrotic bone in the defects treated with Material C and in the control defects between the means at 15 (Material C; 9.28±0.54, Control; 13,48±0,20) and 30 days (Material C; 3.99±0.71, Control; 9.00±0.27) (p=0.032) (p<0.05). Compared with 15th days, statistically significantly fewer amounts of necrotic bone were seen in all defects on the 45th day (p=0.000) (p<0.001). The control defects showed higher amount of necrotic bone than the other treatments at 45th day; however, the NB% differences among treatments were not statistically significant (p>0.05).

The grafted defects with Material A and Material B showed significantly decreased amounts of connective tissue at the 45th day (Material A; 2.73±0.67, Material B; 5.99±1.09) compared to 15th day (Material A; 16.5±0.34, Material B; 23.48±0.67) and 30th day (Material A; 13.66±1.11, Material B; 6.76±0.20) (p=0.00) (p<0.001). Statistically significant decline of amount of connective tissue was also detected at the 45th day (32.03±0.59) compared to 15th day (50.43±0.56) and 30th day (32.5±0.30) in the defects filled with Material C (p=0.034) (p<0.05). The control defects and grafts filled with Material C showed significantly higher amounts of connective tissue than the other defects in all evaluated periods (p=0.000) (p<0.001).

Bone graft particles were gradually resorbed and replaced by newly formed bone. The percentages of residual graft materials decreased within the evaluation time; there were statistically significant reductions on the 45th (Material A; 1.9±0.27, Material B; 2.73±0.46, Material C; 1.6±0.85, Control; 0.00±0.00) day compared with the 15th day (Material A; 21.75±0.80, Material B; 18.1±0.54, Material C; 14.6±0.56, Control; 0.00±0.00) (p=0.05) (p<0.001) and on the 30th day compared with the 15th day (p=0.00) (p<0.05). The percentage of residual graft was significantly lower in Material C group than Material A and B on the 15th day and 30th day (Material A; 4.97±0.89, Material B; 5.72±0.72, Material C; 2.65±0.26, Control; 0.00±0.00) (p=0.006) (p<0.05).

## Discussion

The objective of this study was to evaluate the bone regenerative effect of eggshell-derived graft substitute placed into intrabony defects in rat jaw bones. In addition, we compared the effects of the gel and particle forms of the calcium carbonate graft substitute on bone regeneration. Particle grafts were combined with carrageenan or xanthan gum for the gel matrix.

Various materials based on calcium carbonate have been widely used in dentistry as bone-graft materials to induce healing in bone defects. For example, avian and/or ostrich eggshell has been recently introduced in the field of dentistry (14-18,20,22,25). In this study, hen’s eggshell was proposed as a graft material due to its biocompatibility; the chemical contents of eggshell-derived CaCO3 are likely to host the chemical structure of bone as well.

The eggshell-derived graft materials were reported as resorbable implants in previous studies. In a one year follow-up study, it was reported that eggshell-derived graft material had complete resorption and kinetic degradation capacities depending on its particular size.14 Durmuş et al. reported that smaller and rapidly resorbed powder particles had a low level of bone formation, whereas larger particles seemed to have more osteogenic effects on the sixth month of the experiment period (18). In the present study, we used 1,000 µm particle sized material, which might have been the factor that increased new bone formation in the defect sites.

Various particle sizes of ostrich eggshell were evaluated by Dupoirieux et al., who concluded that 50-µm particles were radiologically undetectable by the first month while 75-µm particles had completely resorbed by the second month. In contrast, 150-300 µm particles were progressively resorbed by the fourth month post-implantation (14). In this study, minimal amounts of residual graft material were observed on the 45th day of the experimental period, which corroborates results from the previous studies. Further studies may focus on critical particle sizes and their osteoinductive effects.

Major expectations from a bone graft material involve enhanced wound healing and induction of new bone formation (2,6,7,26). The results of the present study demonstrated neither the evidence of inflammation nor the presence of necrotic bone tissue in eggshell-derived graft material-treated defects when compared to the control on the 45th day post-implantation. Therefore, eggshell-derived graft material may be accepted as a biocompatible material that can enhance wound healing. Furthermore, significantly higher levels of osteoid formation in the eggshell grafted defects and complete defect filling were found with Material A. These findings are consistent with the results of other studies, which reported good biocompatibility and improved bone formation with calcium carbonate graft materials (14,17,18,22).

One of the aims of the present study was to determine the osteoinductive properties of the eggshell graft materials. Previous studies demonstrated a lack of osteoinduction in defect areas treated with eggshell-derived bone substitute (17,15,20).

However, the material is not said to be osteoinductive, as Baliga et al. (16) suggested that hen’s eggshell powder enhanced bone regeneration in the defect margins. Although the regeneration was confined to the defect margins, Durmuş et al. have reported limited bone regeneration on the 90th day after grafting with ostrich eggshell-derived graft materials and eggshell membranes (20). In addition, surfaces of the particles were localized in deeper regions of the defect sites. However, Dupoirieux et al.(14) presented data regarding the mechanism of osseointegration around ostrich implants in a pilot in vivo study.

In the present study, eggshell-grafted defects showed a greater amount of regenerated bone compared to unfilled defects at healing periods. These findings are inconsistent with the results from other studies (14,17,18,22). Increased amounts of mineralized new bone displaying biodegradable behaviors can be expected by the replacement of graft particles with newly formed bone. Material A particles showed the most powerful osteoproductive activity, with Material B showing next highest levels. Osteoid formation was localized to the defect margins and the deeper regions of the defect sites. The treatment of powder graft material with carrageenan or xanthan gum might have stimulated the biologically active molecules at the onset of osteogenic activity of the particles. However, the osteoproductive activities of the graft materials are associated with several factors such as the structure of the graft material, species of the animal and surface area of the bone (27). For this reason, further studies are warranted to confirm hypotheses involving the osteoinductive properties of eggshells.

It has been suggested that calcium carbonate was an effective biocompatible and resorbable material when implanted in bone tissue (14,18,22), which is supported by the results of the present study. The results of the study demonstrated that the amount of the graft remnants was decreased gradually between the 15th and 45th days with eggshell-derived graft material being replaced with new bone over the same duration. These findings were consistent with results from other studies (14,15,17,18).

Previous studies have shown that eggshell particles are not likely to have immunogenic reactions (17-20,22). In this study, there was no evidence of a hypersentivity reaction against the eggshell-derived graft material, which was also supported by histopathological evaluations with no evidence of eosinophils in the inflammatory infiltrates of the graft treated defects at any time period. In addition, excessive foreign body reaction, dense neutro-philic infiltration, necrotic tissue or giant cells were not observed in any subject except for one. These findings may reveal the biocompatibility of the eggshell powder and gel as graft materials.

The inflammatory response in the groups treated with eggshell-derived graft material was not higher than in the controls on the 45th day. Dupoirieux et al.(14) and Durmuş et al. (18) observed mild inflammatory reactions to ostrich eggshell particles in the first month, with inflammation decreasing progressively at later stages. The hen eggshell-derived gel form graft material that was prepared under these conditions might be considered as biocompatible depending on the results from the present study.

Although our study has some limitations (e.g., number of subjects and study period), it can be concluded that eggshell gel or powder in intrabony defects are potential candidate graft materials. Further studies are needed to assess the application of the materials in clinical settings.
